# Evidence for a Rad18-Independent Frameshift Mutagenesis Pathway in Human Cell-Free Extracts

**DOI:** 10.1371/journal.pone.0036004

**Published:** 2012-04-27

**Authors:** Régine Janel-Bintz, Jérôme Wagner, Lajos Haracska, Marcia Chia Miao Mah-Becherel, Marc Bichara, Robert P. Fuchs, Agnès M. Cordonnier

**Affiliations:** 1 Université de Strasbourg, UMR7242 Biotechnologie et Signalisation Cellulaire, Ecole Supérieure de Biotechnologie de Strasbourg, Illkirch, France; 2 Institute of Genetics, Biological Research Centre, Hungarian Academy of Sciences, Szeged, Hungary; 3 Campus J. Aiguier, UPR3081 Genome Instability and Carcinogenesis, Marseille, France; University of Massachusetts Medical School, United States of America

## Abstract

Bypass of replication blocks by specialized DNA polymerases is crucial for cell survival but may promote mutagenesis and genome instability. To gain insight into mutagenic sub-pathways that coexist in mammalian cells, we examined N-2-acetylaminofluorene (AAF)-induced frameshift mutagenesis by means of SV40-based shuttle vectors containing a single adduct. We found that in mammalian cells, as previously observed in *E. coli*, modification of the third guanine of two target sequences, 5'-GGG-3' (3G) and 5'-GGCGCC-3' (NarI site), induces –1 and –2 frameshift mutations, respectively. Using an *in vitro* assay for translesion synthesis, we investigated the biochemical control of these events. We showed that Pol eta, but neither Pol iota nor Pol zeta, plays a major role in the frameshift bypass of the AAF adduct located in the 3G sequence. By complementing PCNA-depleted extracts with either a wild-type or a non-ubiquitinatable form of PCNA, we found that this Pol eta-mediated pathway requires Rad18 and ubiquitination of PCNA. In contrast, when the AAF adduct is located within the NarI site, TLS is only partially dependent upon Pol eta and Rad18, unravelling the existence of alternative pathways that concurrently bypass this lesion.

## Introduction

Translesion synthesis (TLS) is a regulated and coordinated process in which the replacement of the high fidelity replicative DNA polymerase by one or several specialized TLS polymerases allows replication through DNA blocking lesions that have escaped repair. Human TLS DNA polymerases include Y-family polymerases –DNA polymerase eta (Polη), DNA polymerase iota (Polι), DNA polymerase kappa (Polκ), and Rev1- and one B-family polymerase, DNA polymerase zeta (Polζ) that cooperate to deal with the vast diversity of DNA damages [1]. In humans, loss of Pol eta, required for accurate replicative bypass of cyclobutane pyrimidine dimers induced by UV radiation, results in the variant form of *Xeroderma pigmentosum* (XPV) [2], [3].

The Rad6/Rad18 dependent monoubiquitination of PCNA (Proliferating Cell Nuclear Antigen), sliding clamp associated to the DNA polymerases, is critical for TLS regulation [4], [5], [6], [7]. In addition, eukaryotic TLS polymerases are regulated by posttranslational modifications [8], [9], [10], [11] that may modulate their interactions with specific protein partners.

It is clear that different sub-pathways of TLS coexist in eukaryotic cells due to the large variety of lesions and proteins involved. The characterization of proteins required for TLS across a given lesion is a useful approach to precisely understand their regulation. In this paper, we analysed the bypass of the N2-acetyl-aminofluorene (AAF) adduct in mammalian cells. Binding of the AAF on the C8 of guanine residues severely impairs DNA replication by high-fidelity replicative DNA polymerases [12]. DNA synthesis opposite AAF lesions has been shown to involve a combination of specialized DNA polymerases in *E. coli* [13] and *S. cerevisiae* [14], [15], [16]. In addition in *E. coli*, AAF adducts trigger hot spots (i) of –1 frameshift mutations when located at the third G in monotonous runs of G bases (5'-GGG-3' 5'-GG-3') and (ii) of –2 frameshift mutations in regions of alternating GC base pairs, further referred to as the NarI site (5'-GGCGCC-3' 5'-GGCC-3') [17]. Within these two particular DNA sequence contexts, insertion of a cytosine residue opposite the lesion increases the likelihood of forming slipped mispairs that, when elongated, give rise to frameshift products. Despite similar slippage mechanisms, the AAF bypass within these two DNA sequences requires a different set of DNA polymerases in *E. coli* [13]. In this paper, we present data showing that –1 and –2 frameshift mutations are observed in mammalian cells transfected with SV40-based shuttle vectors monomodified on the third guanine of each target DNA sequence. Using cell-free extracts, we examined the contribution of different translesional DNA polymerases to the bypass within these two sequence contexts. We clearly show that whereas Pol eta, along with Rad18 and monoubiquitinated PCNA, fully mediate the bypass of the AAF adduct located within the 3G sequence, these proteins only partially contribute to the bypass across the same lesion within the NarI site, thus unravelling a yet uncharacterized Rad18-independent TLS pathway.

## Materials and Methods

### Cell lines and culture conditions

Cells were grown at 37°C in Dulbecco's modified Eagle's medium (DMEM) supplemented with 10% foetal calf serum and 1% gentamicin (0.5 mg/ml). MRC5-V1 cells are SV40-transformed normal human lung fibroblasts [18]. COS-7 cells are African green monkey SV40-transformed kidney fibroblasts [19]. The XP30RO cell line (SV40-transformed XPV human fibroblasts) has a homozygous deletion in the Pol eta gene resulting in a truncated protein of only 42 amino acids [3]. XP12RO cells (SV40-transformed XPA human fibroblasts) were cultured in the presence of hygromycin (250 mg/ml) for the maintenance of the plasmid p205-KMT11 encoding the SV40 T antigen [20]. The type I Burkitt's lymphoma BL2 cell lines, in which the genes coding for Rev3, Pol eta, Pol iota or for both Pol eta and Pol iota have been inactivated by homologous recombination, were already described [21]. The human colon carcinoma cell line HCT116-Rad18^KO^ [22] was cultured in the presence of G418 (300 μg/ml) and puromycin (0.3 μg/ml).

**Table 1:** Mutagenic TLS through AAF within the 3G sequence or the NarI site in COS-7 and XP12RO cells.

Plasmids	Nar0	Nar3.AAF	Nar3.AAF	3G0	3G3.AAF	3G3.AAF
AAF location	−	Leading	Lagging	−	Leading	Lagging
Frameshift mutation frequency (10^−5^) in COS-7 cells	<2	21	31	<1	737	1099
Number of colonies in COS-7 cells	0/45,218	12/56,921	20/63,820	0/72,912	485/65,797	487/44,287
Frameshift mutation frequency (10^−5^) in XP12RO cells	<9	17	24	<1	1345	1303
Number of colonies in XP12RO cells	0/10,884	3/17,460	5/20,925	0/98,976	383/28,471	360/27,620

Frequencies of frameshift mutations were expressed as the ratio of the targeted mutant blue colonies to total transformants.

### Construction of the monomodified plasmids

The construction of the AAF monomodified single-stranded plasmids (pUC3G and pUCNar) and the SV40 origin-containing vectors have been described previously [23], [24], [25]. The single AAF adduct is located either on the leading or the lagging strand templates in the early part of the lacZ gene in a +1 (for the pMKB3G and the pMZB3G) or +2 (for the pMKBNar and pMZBNar) reading frame, allowing respectively for –1 or –2 frameshift errors to be detected phenotypically in *E. coli* as blue colonies on Xgal-containing indicator plates.

**Figure 1:**
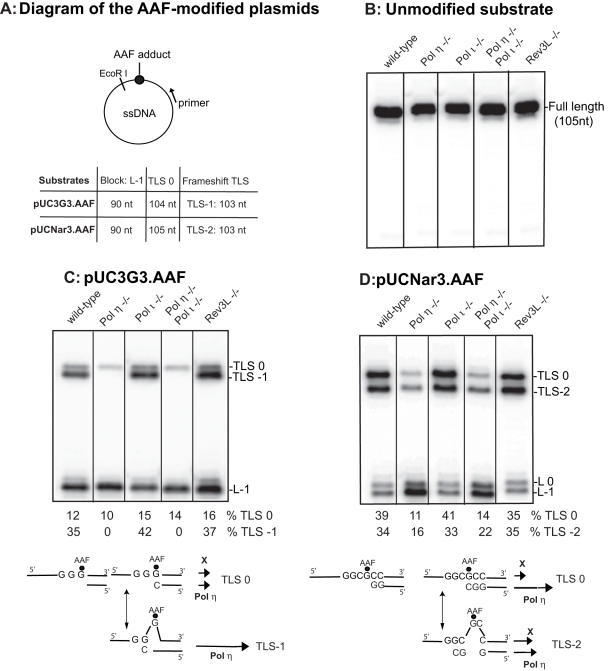
TLS through the G-AAF adduct in the Pol h, Pol i and Rev3 mutant BL2 cell-free extracts. Diagram of the AAF-modified plasmids (panel A). Lengths of the strands produced upon elongation of the labeled primer are indicated; nt, nucleotides. 10 fmoles of unmodified DNA (panel B) or AAF-modified substrates (panels C and D) were incubated 30 min at 37°C in the presence of BL2 cell-free extracts (15 mg) in a final volume of 6.25 ml, as indicated. The samples were analysed by electrophoresis through a 8% denaturing polyacrylamide gel. L-1 and L0 are products generated if synthesis is blocked one nucleotide before and opposite the lesion, respectively. TLS0 and TLS-1 or –2 are products from TLS via non-slipped and slipped intermediates, respectively. Models for the bypass of the AAF lesion in the two sequence contexts are shown below each gel. The length of each arrow is proportional to the involvement of the different factors.

### Transfection and rescue of the replicated plasmids

COS-7 cells were seeded at a density of 5×10^5^ per 100 mm dish, incubated for 24 hours and transfected with plasmid DNA by the calcium phosphate coprecipitation method. Transfection was performed with 50 ng of the AAF-monomodified plasmid shuttle vector and 5 mg carrier plasmid DNA (pBR329). Cells were incubated for 48 hours and then collected for extrachromosomal DNA extraction by the HIRT procedure [26]. At least three independent cell transfections were performed for each plasmid.

### Bacterial transformation and mutant characterization

Plasmid DNA isolated from transfected cells was digested with DpnI to restrict all input plasmid DNA that retained the bacterial methylation pattern and could give rise to white colonies. *E. coli* strains TB1 were transformed by the DpnI-digested plasmids, and transformants were analysed on indicator plates containing 100 mg/ml of X-Gal (5-bromo-4-chloro-3-indolyl b-D-pyranoside) (Roche), 1 mM IPTG (isopropyl b-D-thiogalactopyranoside) (Roche) and 50 mg/ml ampicillin (Sigma). Mutagenesis was detected by means of a colorimetric assay (white to blue) based on a,w-complementation of beta-galactosidase, as the target sequence is located on the *lacZ*' gene of the shuttle vector. Blue mutant colonies were purified by retransformation into TB1. Plasmid DNA from purified blue colonies was recovered by the alkaline lysis method and sequenced. The frequency of –1 or –2 frameshift mutagenesis at the 3G sequence or at the NarI site was calculated as the ratio of –1 or –2 targeted mutant colonies to total transformants, respectively.

### Primer extension analysis

Primer extension analysis were performed in the conditions previously described [27]. The reaction mixture (6.25 ml) containing 10 fmoles of primed circular single-stranded DNA and the whole cell extract (10–30 mg) was incubated at 37°C for 30 min to 1 hour in 50 mM Hepes-KOH (pH 7.9), 7 mM MgCl_2_, 1 mM DTT, 4 mM ATP, 200 mM of dNTPs, 40 mM creatine phosphate, 100 mg/ml creatine kinase. Replication products were digested with EcoRI and PvuII restriction enzymes and analysed by electrophoresis on a 8% polyacrylamide-7 M urea denaturing gel. Quantification of the levels of TLS was determined by Phospho-Imager and Image J analysis. The percentage of TLS was calculated as the ratio of the intensity of the bands of non-slipped TLS (TLS 0) or slipped TLS (TLS –1 or –2) to the sum of the intensity of the TLS, L-1 and L0 bands.

### Preparation of PCNA-depleted extracts

For the depletion of PCNA from cell extracts, we took advantage of the PCNA binding capacities of a previously described peptide, P21 consensus motif 1 (CON1: SAVLQKKITDYFHPKK) [28]. 5' phosphorylated complementary oligos encompassing the DNA sequence coding for this peptide (5'-catggctAGCGCGGTGCTGCAGAAAAAAATTACCGATTATTTTCATCCGAAAAAATAATAAggtac-3' and 5'-cTTATTATTTTTTCGGATGAAAATAATCGGTAATTTTTTTCTGCAGCACCGCGCTagc-3'; Sigma-Aldrich) were annealed and ligated to NcoI – KpnI digested pETM-30 scaffold (Invitrogen) to generate the pET-CON1 vector expressing a 6His-GST-CON1 peptide fusion. The peptide was over-expressed in BL21 *E. coli* cells and further purified by metal chelate affinity chromatography using a BioLogic system (Biorad) and HisTrap HP column (GE Healthcare). The purified fusion peptide was then immobilized on Dynabeads His-Tag paramagnetic beads (Invitrogen) according to the manufacturer's instructions (500 µg of peptide per 3 mg of beads). After extensive washes, the beads were mixed with 5 mg of cellular extracts in 850 ml of DIA buffer (100 mM potassium glutamate, 30 mM HEPES pH 7.9, 1 mM DTT, 10% glycerol) containing 50 mM NaCl and incubated for 1 hour at 4°C with continuous rotation. After magnetic separation, the supernatant was filtered through Ultrafree MC 0.45 µm HV Durapore filters (Millipore). The efficiency of the depletion was checked by Western blotting.

**Figure 2:**
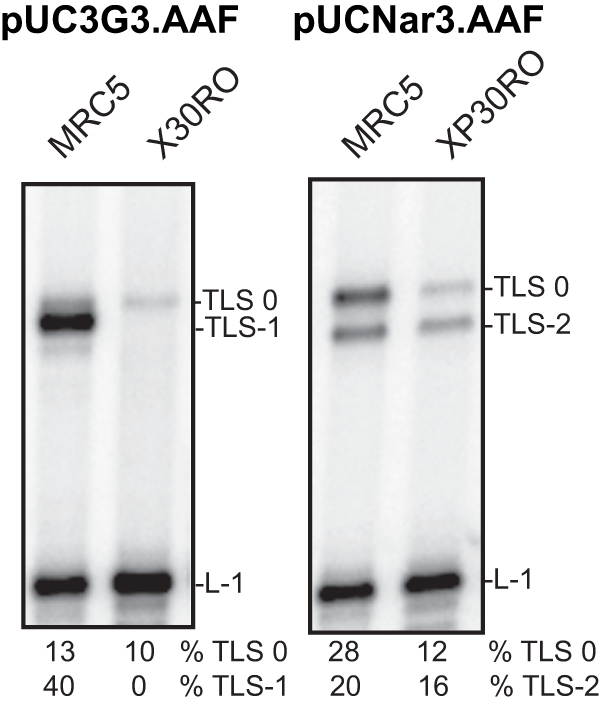
TLS through the G-AAF adduct in MRC5 and XP30RO cell-free extracts. MRC5 (WT) and XP30RO (Pol eta deficient) cell-free extracts (20 mg) were incubated 30 min at 37°C in the presence of 10 fmoles of AAF-modified substrates either at the 3G sequence or at the NarI site in a final volume of 6.25 ml, as indicated. The samples were analysed by electrophoresis through a 8% denaturing polyacrylamide gel. L-1 is a product generated if synthesis is blocked one nucleotide before the lesion. TLS0 and TLS-1 or –2 are products from TLS via nonslipped and slipped intermediates, respectively.

**Figure 3:**
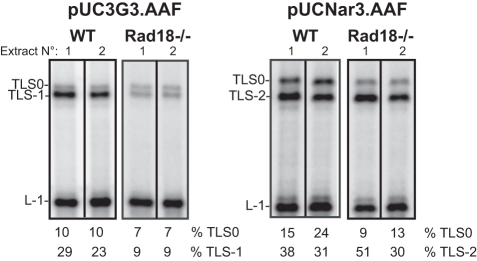
Analysis of Rad18 dependence of the G-AAF bypass in HCT116 cell-free extracts. HCT116 cell-free extracts, wild-type (WT) and Rad18−/− (20 mg), were incubated 30 min at 37°C in the presence of 10 fmoles of AAF-modified substrates either at the 3G sequence or at the NarI site in a final volume of 6.25 ml, as indicated. The samples were analysed by electrophoresis through a 8% denaturing polyacrylamide gel. L-1 is a product generated if synthesis is blocked one nucleotide before and opposite the lesion, respectively. TLS0 and TLS-1 or –2 are products from TLS via non-slipped and slipped intermediates, respectively.Quantitative analysis of experiments with two independent extracts are presented.

## Results and Discussion

### AAF-induced frameshift mutagenesis in mammalian cells

We designed two families of shuttle vectors containing a site-specific AAF adduct on the third G in either a run of three guanines (5'-GGG^AAF^-3', referred to as 3G3.AAF) or an alternating GC sequence (5'-GGCG^AAF^CC-3', referred to as Nar3.AAF) located within the *lacZ*’ target gene. The SV40 origin-containing plasmids were transfected in COS-7 cells, and after 48 hours incubation of the cells, the plasmid DNA was isolated and transformed into *E. coli* in order to phenotypically detect frameshift mutations.

**Figure 4:**
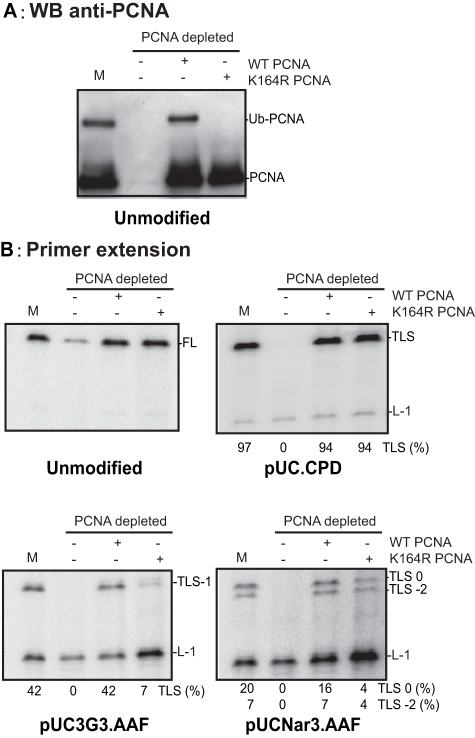
Analysis of Ub-PCNA dependence of G-AAF bypass in MRC5 cell-free extracts. Mock depleted (M) or PCNA depleted MRC5 cell extracts (20 mg) were incubated 30 min at 37°C in the presence of 10 fmoles of unmodified or modified substrates in a final volume of 6.25 ml, as indicated. Recombinant wild-type (WT) or mutated K164R PCNA (60 ng) was added to the reactions, as indicated. Aliquotes of the samples were analysed either by Western blot with an anti-PCNA antibody (panel A) or by 8% denaturing polyacrylamide gel electrophoresis (panel B). L-1 is a product generated if synthesis is blocked one nucleotide before the lesion. TLS0 and TLS-1 or –2 are products from TLS via non-slipped and slipped intermediates, respectively. FL are Full Length products.

In COS-7 cells transfected with the unmodified plasmids, the spontaneous mutation frequency is less than 10^−5^ (Table 1). AAF modification at the third G increases the –2 frameshift mutation frequency at the NarI site to about 2×10^−4^, and the –1 frameshift mutation frequency at the three G run to about 10^−2^ (Table 1). We did not observe any blue mutant colony upon transfection of vectors modified on the first or the second G of the NarI sequence (among 80,409 transformants for Nar1.AAF and 191,128 transformants for Nar2.AAF; data not shown), demonstrating that only the presence of the adduct at the third G is critical to induce frameshift mutagenesis. The relatively low frequency of –2 frameshift mutagenesis compared to the –1 frameshift mutagenesis was also found upon transfection in the repair-deficient human cell line XP12RO (XPA; Table 1), showing that the higher mutagenicity of the AAF adduct located at the 3G sequence does not result from a preferential repair of the Nar3.AAF substrate. It should be stressed that the overall contribution of TLS to the AAF bypass in mammalian cells is potentially much higher as only frameshift TLS events are scored in this assay. In both sequence contexts, the AAF-induced mutation frequency is not significantly different whether the adduct is located on the leading or the lagging strand (Table 1). This result is consistent with similar studies that monitor TLS through CPD lesions [29], 6–4 photoproducts [30], or a thymine glycol [31] in human cells using equivalent assays. Collectively, these observations suggest that similar genetic mechanisms contribute to TLS, irrespective of the location of the lesion on the leading or the lagging DNA strand of an episomal plasmid.

In *E. coli*, it has been shown that a different set of DNA polymerases is involved in the bypass of the AAF adduct located within the two different sequence contexts analysed here, Pol II (a B-family DNA polymerase) being exclusively required for –2 frameshift mutagenesis, whereas Pol V (a Y-family DNA polymerase) is involved in –1 frameshift mutagenesis [13]. In contrast in *S. cerevisae*, the frameshift mutagenesis induced by AAF adducts within both repetitive sequences depends on Pol zeta [15]. These differences between organisms and the high –1 mutation frequency obtained in mammalian cells compared to the –2 mutation frequency prompted us to further investigate the biochemical control of these two pathways.

### Evidence for a Pol eta-independent TLS pathway

We have previously shown that extracts from wild-type primary or transformed cells were able to perform TLS through an AAF adduct located at the 3G sequence 10-fold more efficiently than extracts from XPV cells, indicating that DNA Pol eta is involved in the reaction [27], [32]. In order to evaluate the role of this DNA polymerase and of possible other TLS polymerases (in particular the B-family polymerase Pol zeta) in the bypass of AAF adducts within the two sequence contexts analysed in this paper, we conducted similar experiments with extracts from BL2 cells knocked-out for Pol eta and Pol iota (either singly or in combination), and for REV3L (the catalytic subunit of Pol zeta) [21]. We measured the capacity of the cell-free extracts to extend a primer past an AAF adduct located at the third G in the 3G or NarI sequences on a circular single-stranded DNA plasmid (Fig. 1A). This approach allowed us to analyse the partitioning between non-slipped (TLS0) and slipped products (TLS-1 or TLS-2).

As shown in figure 1B, the different extracts display identical DNA synthesis activities when tested with a primed unmodified single-stranded template. In the presence of an AAF adduct, the patterns of the elongation products indicate that extracts from wild-type cells are able to bypass the AAF lesion efficiently in either sequence context. When the AAF adduct is located in the run of 3G (Fig. 1C), elongation from the slipped intermediate (TLS-1) catalysed by extracts from wild-type cells largely predominates over the non-slipped elongation reaction (TLS0). TLS0 is slightly reduced and TLS-1 totally abolished in the absence of Pol eta, as observed for extracts from primary human fibroblasts [27], [33]. No marked difference was detected between normal cell extracts and those that contain neither Pol iota nor Rev3, demonstrating that these two specialized DNA polymerases do not play any significant role *in vitro* in TLS through the AAF adduct in this sequence context. We are aware that we cannot totally exclude any participation of Rev3 in the bypass, as we have not checked the TLS activity in the double mutant cell extract, Pol eta/ Rev3.

When the AAF adduct is located within the NarI sequence, similar quantities of full length elongation products (TLS0) and two bases slippage products (TLS-2) were observed upon incubation with extracts from wild-type cells (Fig. 1D). The amount of both TLS0 and TLS-2 decreased in Pol eta deficient extracts, demonstrating that Pol eta is involved in the synthesis of both products. A significant amount of residual TLS was observed in extracts from the single Pol eta mutant cells as well as in the double Pol eta/ Pol iota mutant cells, strongly suggesting the involvement of at least another DNA polymerase able to perform both types of TLS through the AAF adduct in the NarI context. Deletion of the rev3 coding sequence did not significantly change the amount of the TLS products, indicating that Pol zeta does not play a major role in the bypass of the AAF lesion in the NarI sequence, as in the 3G sequence.

To rule out the possibility that these observations are specific to the lymphoma cell type used, we extended our experiments by comparing the TLS activities of extracts from either normal (MRC5) or XPV (XP30RO:Pol eta deficient) transformed human fibroblasts. The data clearly show that also in fibroblastic cell lines, the defect in Pol eta results in the absence of TLS-1 on the pUC3G3.AAF substrate and a strong reduction of both TLS0 and TLS-2 on the pUCNar3.AAF substrate (Fig. 2).

Altogether, the data presented here show that in human cell extracts Pol eta, but not Pol zeta, plays a major role in the bypass of AAF adducts located within the two sequence contexts analyzed. In *S. cerevisiae*, this lesion is bypassed in a two-polymerase mechanism, Pol eta being involved at the insertion step whereas Pol zeta is required at the extension step [15], [16]. Differences in the biochemical properties of the human as compared to the yeast Pol eta may account for this divergence. Indeed, although both enzymes are efficient in incorporating a C opposite the G-AAF adduct, only the human Pol eta can continue the chain elongation [34], [35].

These results show that distinct pathways coexist in human cells to bypass the AAF adduct. One of these pathways involves Pol eta, which is required for all –1 frameshift mutagenesis events at the 3G sequence, and for a significant proportion of both TLS0 and TLS-2 frameshift mutagenesis at the NarI sequence. In addition, at least another, yet uncharacterized pathway (X-dependent) is responsible for some part of the TLS0 at the 3G sequence and for a sizable fraction of both TLS0 and TLS-2 at the NarI site. Deciphering this(ese) alternative pathway(s), which also exist in extracts from primary or transformed XPV fibroblasts (data not shown), may be of major importance in order to get a better understanding of mutagenesis in human cells.

### Evidence for a Rad18, Ub-PCNA-independent TLS pathway

Using cell-free extracts, we have previously established that PCNA is mono-ubiquitinated in a Rad18-dependent manner during a primer extension reaction when the replication complex encounters a pausing site on a single-stranded DNA template [32]. Furthermore, TLS through an AAF adduct located on the first G of the 3G sequence was found to be dependent upon Rad18 [36]. Similarly, we show here that in Rad18 knock-out cell extracts, the efficiency of TLS-1, which exclusively depends on Pol eta activity is drastically reduced as compared to that of wild-type cell extracts (Fig. 3). Surprisingly, when we used the pUC.Nar3AAF substrate in the same assay, the depletion of Rad18 resulted in a decrease in TLS0 without affecting TLS-2 (Fig. 3), indicating that Rad18 by itself or Ub-PCNA (or both) are not absolutely required for the production of TLS-2 at the NarI site, thus uncovering a Rad18-independent frameshift mutagenesis pathway in mammalian cells. It has to be noted that upon incubation with pUCNar3.AAF, the HCT116 cell extract shows a slightly higher TLS-2 efficiency than BL2 (Fig. 1) and MRC5 (Fig. 2) cell extracts. These data suggest that distinct cell types may express different amounts of factors involved in each specific pathway.

Whether Rad18 contributes to TLS in cell-free extracts by interacting with Pol eta or by triggering PCNA ubiquitination remains uncertain. In order to examine more directly the role of Ub-PCNA in the bypass of the AAF adduct, we performed the elongation reactions in a cell extract, first depleted for PCNA and then complemented with either wild-type or K164R mutated PCNA. We exploited the very strong and specific interaction between PCNA and a peptide derived from p21 [28] to deplete PCNA nearly to completion in MRC5 cell-free extracts (Fig. 4A). As expected, endogenous PCNA and purified wild-type PCNA, but not K164R PCNA, were monoubiquitinated during the primer extension reaction (Fig. 4A). Both wild-type and K164R purified PCNA were able to restore efficient primer extension on an unmodified substrate, indicating that essential proteins have not been co-depleted together with PCNA (Fig. 4B). Both wild-type and K164R PCNA were equally able to rescue the bypass of the CPD lesion (Fig. 4B), demonstrating unambiguously that the ubiquitylation-defective PCNA mutant is a functionally proficient sliding clamp able to promote TLS through this UV-induced lesion, consistent with another *in vitro* study [37]. This result is in agreement with a model in which Pol eta can be recruited at the site of the damage in the absence of Ub-PCNA in human fibroblasts [38]. We have previously shown [36] that the PCNA interacting peptide (PIP) motif of Pol eta is required for efficient bypass of CPD in this system. Hence, interaction of Pol eta with PCNA, and possibly with other proteins, is sufficient to stabilize the TLS polymerase during CPD bypass. Recently TLS across a TT CPD has been shown to occur in mouse embryo fibroblasts expressing PCNA K164R [39], albeit at reduced extent than in cells expressing wild type PCNA. Furthermore, Pol eta provides a critical advantage for UV survival and replication blocks recovery in mouse embryo fibroblasts expressing PCNA K164R [39], [40]. Collectively, these *in vivo* data show that Pol eta activation may occur independently of Ub-PCNA.

On the contrary, K164R PCNA is not able to promote TLS through the AAF adduct located at the 3G sequence (Fig. 4B), pointing to the importance of the posttranslational modification of PCNA at this site for the bypass of the AAF adduct by Pol eta, presumably by increasing its residence time [38].

Unexpectedly, when the AAF adduct was located in the NarI site, the ubiquitylation-defective mutant K164R PCNA partially rescued TLS0, and even more TLS-2. Hence, in this sequence context, an as yet uncharacterized pathway is able to generate TLS0 and TLS-2 products in a Rad18 and Ub-PCNA independent manner.

### Conclusions and perspectives

In this paper, we show that in mammalian cells, the protein requirement for the bypass of an AAF adduct within two different sequence contexts fundamentally differ.

TLS through the AAF adduct in the 3G sequence is mediated by DNA polymerase eta and requires Rad18 and Ub-PCNA. The high proportion of slipped TLS observed *in vitro* presumably accounts for the high frequency of –1 frameshift mutagenesis observed *in vivo* upon transfection of mamalian cells with the 3G3 monomodified plasmid. In comparison, the frequency of –2 frameshift observed upon transfection with the Nar3 monomodified plasmid is low and does not correlate with the amount of TLS-2 observed *in vitro*. *In vivo*, the bypass of a DNA damage triggers many different responses among which TLS is only one of them. Damage avoidance pathways that are not active in our *in vitro* assay may contribute more efficiently *in vivo*, to the bypass of the AAF lesion within the NarI sequence than within the 3G sequence. Given the specific signature of Pol eta, relative to the AAF-induced mutations within the 3G sequence, the corresponding AAF monomodified plasmid provides a useful tool to investigate the regulation of this DNA polymerase in human cells and to examine the impact of different mutations in the protein. Bypass of the AAF adduct was achieved in non-irradiated cells, indicating that ubiquitination of PCNA and recruitment of Pol eta does not depend on global checkpoint activation, but on cis-acting factors at the blocked replication fork. In addition to the Pol eta and Ub-PCNA dependent pathway, the bypass of the AAF adduct positioned within the NarI site is partially dependent upon another, as yet uncharacterized pathway that does not involve Rad18. One important goal will be to identify the DNA polymerase(s) involved in this pathway and to elucidate its regulation.

## References

[1] Prakash S, Johnson RE, Prakash L (2005). Eukaryotic translesion synthesis DNA polymerases: specificity of structure and function.. Annual review of biochemistry.

[2] Johnson RE, Prakash S, Prakash L (1999). Efficient bypass of a thymine-thymine dimer by yeast DNA polymerase, Poleta.. Science.

[3] Masutani C, Kusumoto R, Yamada A, Dohmae N, Yokoi M (1999). The XPV (xeroderma pigmentosum variant) gene encodes human DNA polymerase eta.. Nature.

[4] Hoege C, Pfander B, Moldovan GL, Pyrowolakis G, Jentsch S (2002). RAD6-dependent DNA repair is linked to modification of PCNA by ubiquitin and SUMO.. Nature.

[5] Stelter P, Ulrich HD (2003). Control of spontaneous and damage-induced mutagenesis by SUMO and ubiquitin conjugation.. Nature.

[6] Kannouche PL, Wing J, Lehmann AR (2004). Interaction of human DNA polymerase eta with monoubiquitinated PCNA: a possible mechanism for the polymerase switch in response to DNA damage.. Molecular cell.

[7] Watanabe K, Tateishi S, Kawasuji M, Tsurimoto T, Inoue H, Yamaizumi M (2004). Rad18 guides poleta to replication stalling sites through physical interaction and PCNA monoubiquitination.. EMBO J.

[8] Chen YW, Cleaver JE, Hatahet Z, Honkanen RE, Chang JY (2008). Human DNA polymerase eta activity and translocation is regulated by phosphorylation.. Proceedings of the National Academy of Sciences of the United States of America.

[9] Pages V, Santa Maria SR, Prakash L, Prakash S (2009). Role of DNA damage-induced replication checkpoint in promoting lesion bypass by translesion synthesis in yeast.. Genes & development.

[10] Bienko M, Green CM, Sabbioneda S, Crosetto N, Matic I (2010). Regulation of translesion synthesis DNA polymerase eta by monoubiquitination.. Molecular cell.

[11] Gohler T, Sabbioneda S, Green CM, Lehmann AR (2011). ATR-mediated phosphorylation of DNA polymerase eta is needed for efficient recovery from UV damage.. The Journal of cell biology.

[12] Belguise-Valladier P, Fuchs RP (1995). N-2-aminofluorene and N-2 acetylaminofluorene adducts: the local sequence context of an adduct and its chemical structure determine its replication properties.. Journal of molecular biology.

[13] Napolitano R, Janel-Bintz R, Wagner J, Fuchs RP (2000). All three SOS-inducible DNA polymerases (Pol II, Pol IV and Pol V) are involved in induced mutagenesis.. EMBO J.

[14] Baynton K, Bresson-Roy A, Fuchs RP (1998). Analysis of damage tolerance pathways in Saccharomyces cerevisiae: a requirement for Rev3 DNA polymerase in translesion synthesis.. Molecular and cellular biology.

[15] Baynton K, Bresson-Roy A, Fuchs RP (1999). Distinct roles for Rev1p and Rev7p during translesion synthesis in Saccharomyces cerevisiae.. Molecular microbiology.

[16] Bresson A, Fuchs RP (2002). Lesion bypass in yeast cells: Pol eta participates in a multi-DNA polymerase process.. The EMBO journal.

[17] Fuchs RP, Schwartz N, Daune MP (1981). Hot spots of frameshift mutations induced by the ultimate carcinogen N-acetoxy-N-2-acetylaminofluorene.. Nature.

[18] Huschtscha LI, Holliday R (1983). Limited and unlimited growth of SV40-transformed cells from human diploid MRC-5 fibroblasts.. Journal of cell science.

[19] Gluzman Y (1981). SV40-transformed simian cells support the replication of early SV40 mutants.. Cell.

[20] Stary A, Sarasin A (1992). Simian virus 40 (SV40) large T antigen-dependent amplification of an Epstein-Barr virus-SV40 hybrid shuttle vector integrated into the human HeLa cell genome.. The Journal of general virology 73 ( Pt.

[21] Gueranger Q, Stary A, Aoufouchi S, Faili A, Sarasin A (2008). Role of DNA polymerases eta, iota and zeta in UV resistance and UV-induced mutagenesis in a human cell line.. DNA repair.

[22] Shiomi N, Mori M, Tsuji H, Imai T, Inoue H (2007). Human RAD18 is involved in S phase-specific single-strand break repair without PCNA monoubiquitination.. Nucleic acids research.

[23] Veaute X, Fuchs RP (1993). Greater susceptibility to mutations in lagging strand of DNA replication in Escherichia coli than in leading strand.. Science.

[24] Napolitano RL, Fuchs RP (1997). New strategy for the construction of single-stranded plasmids with single mutagenic lesions.. Chemical research in toxicology.

[25] Thomas DC, Veaute X, Fuchs RP, Kunkel TA (1995). Frequency and fidelity of translesion synthesis of site-specific N-2-acetylaminofluorene adducts during DNA replication in a human cell extract.. The Journal of biological chemistry.

[26] Hirt B (1967). Selective extraction of polyoma DNA from infected mouse cell cultures.. J Mol Biol.

[27] Cordonnier AM, Lehmann AR, Fuchs RP (1999). Impaired translesion synthesis in xeroderma pigmentosum variant extracts.. Molecular and cellular biology.

[28] Zheleva DI, Zhelev NZ, Fischer PM, Duff SV, Warbrick E (2000). A quantitative study of the in vitro binding of the C-terminal domain of p21 to PCNA: affinity, stoichiometry, and thermodynamics.. Biochemistry.

[29] Yoon JH, Prakash L, Prakash S (2009). Highly error-free role of DNA polymerase eta in the replicative bypass of UV-induced pyrimidine dimers in mouse and human cells.. Proceedings of the National Academy of Sciences of the United States of America.

[30] Yoon JH, Prakash L, Prakash S (2010). Error-free replicative bypass of (6–4) photoproducts by DNA polymerase zeta in mouse and human cells.. Genes & development.

[31] Yoon JH, Bhatia G, Prakash S, Prakash L (2010). Error-free replicative bypass of thymine glycol by the combined action of DNA polymerases kappa and zeta in human cells.. Proceedings of the National Academy of Sciences of the United States of America.

[32] Schmutz V, Wagner J, Janel-Bintz R, Fuchs RP, Cordonnier AM (2007). Requirements for PCNA monoubiquitination in human cell-free extracts.. DNA Repair (Amst).

[33] Broughton BC, Cordonnier A, Kleijer WJ, Jaspers NG, Fawcett H (2002). Molecular analysis of mutations in DNA polymerase eta in xeroderma pigmentosum-variant patients.. Proceedings of the National Academy of Sciences of the United States of America.

[34] Masutani C, Kusumoto R, Iwai S, Hanaoka F (2000). Mechanisms of accurate translesion synthesis by human DNA polymerase eta.. The EMBO journal.

[35] Yuan F, Zhang Y, Rajpal DK, Wu X, Guo D (2000). Specificity of DNA lesion bypass by the yeast DNA polymerase eta.. The Journal of biological chemistry.

[36] Schmutz V, Janel-Bintz R, Wagner J, Biard D, Shiomi N (2010). Role of the ubiquitin-binding domain of Poleta in Rad18-independent translesion DNA synthesis in human cell extracts.. Nucleic acids research.

[37] Nikolaishvili-Feinberg N, Jenkins GS, Nevis KR, Staus DP, Scarlett CO (2008). Ubiquitylation of proliferating cell nuclear antigen and recruitment of human DNA polymerase eta.. Biochemistry.

[38] Sabbioneda S, Gourdin AM, Green CM, Zotter A, Giglia-Mari G (2008). Effect of proliferating cell nuclear antigen ubiquitination and chromatin structure on the dynamic properties of the Y-family DNA polymerases.. Molecular biology of the cell.

[39] Hendel A, Krijger PH, Diamant N, Goren Z, Langerak P (2011). PCNA ubiquitination is important, but not essential for translesion DNA synthesis in mammalian cells.. PLoS genetics.

[40] Krijger PH, van den Berk PC, Wit N, Langerak P, Jansen JG (2011). PCNA ubiquitination-independent activation of polymerase eta during somatic hypermutation and DNA damage tolerance.. DNA repair.

